# Printed Flexible Microelectrode for Application of Nanosecond Pulsed Electric Fields on Cells

**DOI:** 10.3390/ma12172713

**Published:** 2019-08-24

**Authors:** Martin Schubert, Jens Rasche, Mika-Matti Laurila, Tiina Vuorinen, Matti Mäntysalo, Karlheinz Bock

**Affiliations:** 1 Electronics Packaging Laboratory, Technische Universität Dresden, 01069 Dresden, Germany; 2 Centre for Tactile Internet with Human-in-the-Loop (CeTI), Technische Universität Dresden, 01062 Dresden, Germany; 3 Electrical Engineering, Faculty of Information Technology and Communication Sciences, Tampere University, 33720 Tampere, Finland

**Keywords:** pulsed electric field treatment, electropermeabilization, flexible electronics, printed electronics, E-jet printing

## Abstract

Medical treatment is increasingly benefiting from biomedical microsystems, especially the trending telemedical application. A promising modality for tumor therapy showed the application of nanosecond pulsed electric fields (nsPEF) on cells to achieve nanoporation, cell death, and other cell reactions. A key technology for this method is the generation of pulsed fields in the nanosecond range with high-field strengths in the range of several kilovolts per centimeter. For further biomedical applications, state-of-the-art setups need to decrease in size and improve their capability of integration into microsystems. Due to demanding electronic requirements, i.e., using high voltages and fast pulses, miniaturization and low-cost fabrication of the electrode is first considered. This paper proposes a proof-of-concept for a miniaturized printed flexible electrode that can apply nsPEF on adherent fibroblast cells. The interdigital gold electrode was printed on polyimide with line-width of about 10 µm using an electrohydrodynamic inkjet printer. Furthermore, an electrical circuit was developed to generate both electrical pulses in the nano-second range and voltages up to 180 V. The electrode was integrated into an experimental setup for in-vitro application to human fibroblasts. Field strengths up to 100 kV/cm with 45 ns pulse duration were applied, depending on the degree of cell confluence. The cells show contraction, detachment from the electrode, and lethal reactions after the nsPEF treatment. Furthermore, this printed miniaturized electrode was found to be suitable for subsequent microsystem integration and further cell experiments to optimize pulse parameters for control of cell reaction and behavior.

## 1. Introduction

Medical cancer treatment depends on the type of cancer; therefore, various methods are continuously under investigation. In addition to the common chemo- and hormonotherapy, radiation therapy and surgical resection [1], other physical methods such as radiofrequency ablation, laser- and microwave-thermal therapy and electrochemotherapy (ECT) have shown full or partial curative treatment in studies [2,3]. The stated therapies are often associated with additional damage to other cells, apart from targeting the tumor itself. ECT is one therapy among the mentioned, which potentially enables a more targeted treatment of only affected cancer cells. It exploits benefits from physical and chemical therapy by enhancing the local application of drugs on tumor cells through electropulsation, if aided with miniaturized and locally applicable electrodes [3,4]. Several clinical trials on different cancer types have reported higher cytotoxicity of drugs on electrically treated tumor cells [3,5,6,7]. A clinical study in 2018 on esophageal cancer reported the feasibility to miniaturize the application of ECT, using a novel endoscopic integrated electrode, therefore offering further enhancement of localized intra-corporal treatment [7].

Electropermeabilization, which is the key mechanism behind ECT, is the increasing permeability of cell membranes on applying short pulsed electric fields [4,8]. Within the lipid bilayer of a cell are membranes pores, formed by physical and chemical mechanisms, which depend on the electrical parameters (pulse duration, number of pulses, amplitude, pulse repetition frequency) applied [3,4,9]. Often, this process is also referred to as electroporation, which in a more-narrow sense is the formation of aqueous pores [4]. Molecular dynamics simulation characterizes pore formation, yet not all permeability processes are fully determined [3,4,10,11]. Electrically-induced permeability of the membrane may stay increased for seconds, minutes or even irreversibly after the pulses. This leads to different cell reactions, such as apoptosis, swelling or rupture [10,11]. In literature, various parameters have been identified as causing electroporation of cell membranes with pulse durations between 1 ns and 1 s and electric field strength between 1 to 300 kV/cm [9,10,12,13,14,15,16]. Generally, the higher the electrical field strength, the more likely is electroporation to be the cause of cell death [17]. For this reason, various research works on different electrode designs have tried to understand the influence of pulse parameters.

The most common biological application for electropermeabilization is the introduction of alien materials into cells wherein the membranes are usually impermeable. In the case of gene transfer, for example, even larger molecules (such as DNA fragments), which are naturally not able to pass the cell membrane, can be introduced [18,19]. In order to treat a large number of cells simultaneously (e.g., within a cell suspension), electroporation cuvettes are normally used, consisting of two capacitor plates. Differently shaped plate electrodes are often applied for extra-corporal or superficial located tumors; needle electrodes are applied for medical application, particularly intra-corporal treatment [7,19,20]. Commercially available devices with macroscopic electrodes that are currently used for treatment need voltages of more than 1000 V in order to achieve the required field [17]. Many scientific approaches for the investigation and development of micro-electroporation electrodes have been performed to lower the required voltage. This can be done, for example, by reducing the pulsing voltage to about 150 V at a feature size of ~100 µm for stationary in-vitro applications [21,22]. A similar approach may also be used for future in-vivo applications (as shown by Huang et al.) with flexible on-skin electrodes [23]. Matsuki et al. highlighted electroporation with longer, low-voltage pulses (75 V/cm, 100 ms) with the advantage of a higher percentage of surviving cells, with respect to the used cell type [24]. There have been approaches to further reduce the application voltage down to 2.5 V for microfluidic devices comprising micropores or nanostructures [17,25,26]. However, additively manufactured electrodes for the application of nsPEF have not yet been described in literature; the electrodes have the potential to lower fabrication costs and reduce waste material. Additive manufacturing technologies on polymeric substrates have the added benefits of individual design and economical usage of biocompatible materials, i.e., gold electrodes. Future in-vivo applications, which often require treatment of large tissue areas, may especially benefit from this approach, in terms of scalable low-pitch microelectrode arrays with individual forms.

This paper aims to demonstrate proof of concept on additively manufactured microelectrodes, together with a self-developed pulse-circuit—a load independent pulse generator for future electroporation experiments with adjustable pulse duration and amplitude. The high-resolution electrohydrodynamic inkjet (E-jet) printing enables the fabrication of interdigital electrode circuitry with approximately 10 times higher resolution (i.e., conductor width/conductor pitch) [27] when compared to conventional piezoelectric inkjet printing [28]. This enables the fabrication of highly miniaturized devices, which offer lower drive voltage and/or higher electric fields for more efficient electroporation. The proposed printed, miniaturized, flexible, biocompatible interdigital electrode is not limited to in-vitro applications. Its biocompatibility, size and flexibility, as well as the fabrication method used enables the electrode to be integrated in implantable microsystems for in-vivo cancer treatment or other selective drug transfer into cells. Furthermore, the approach presented in this paper may also be applied to future medical microsystems, to keep specific areas free of unintended cell adhesion; for instance, the control or treatment of in-stent restenosis as an alternative to drug-eluting stents [29]. Therefore, a stent may be equipped with microelectrodes on foil (comparable with a stent-graft) and nsPEF, applied at the inner walls of the stent. In the future, induced cell death by embedded microelectrodes in surgical instrument surfaces may be considered, for e.g., electrical field force surgery.

## 2. Materials and Methods

### 2.1. Electrodes

The interdigital electrodes were fabricated on a 10-µm-thick polyimide (PI-2611, HD MicroSystems, Hitachi Chemical DuPont MicroSystems L.L.C., Parlin, NJ, USA) foil, which was fabricated using spin coating and cured in nitrogen atmosphere. The electrodes were printed on top of the polyimide substrate with gold nanoparticle ink (Au-Nanometal, ULVAC Ink., Methuen, MA, USA) using an E-jet printer (S050, Super Inkjet Technology Inc., Tsukuba, Japan) with nozzle size SFN (super fine nozzle). The following print parameters were applied: 150 Hz frequency sinusoidal waveform, 0 V bias voltage, 430 V peak-to-peak voltage, 50 µm nozzle-to-substrate distance and 1 mm/s printing speed. Bipolar drive voltage was used to prevent charge accumulation on the substrate and to increase printing stability. Five layers were printed with final electrode thickness of approximately 300 nm and average electrical resistance of 85 ohms after sintering at 250 °C for 1 h. The interdigital electrodes were further connected with inkjet printed (DMP-2831, Fujifilm Dimatix Inc., Santa Clara, CA, USA) silver tracks (NPS-JL, Harima Chemicals Group, Inc., Tokyo, Japan, 10 layers, 55 µm drop spacing, line width 500 µm) to enable an interface to the output of the developed electrical circuit. The flexible electrodes were connected with conductive adhesive (H20E, Epoxy Technology Inc., Billerica, MA, USA) to copper wires outside of the well.

The silver tracks were covered with an additional layer of polyimide to prevent contact with the cells or the cell medium. Only the interdigital gold electrodes, with 1.0 × 1.0 mm^2^ dimensions, were left uncovered to enable uninsulated cell contact.

**Figure 1:**
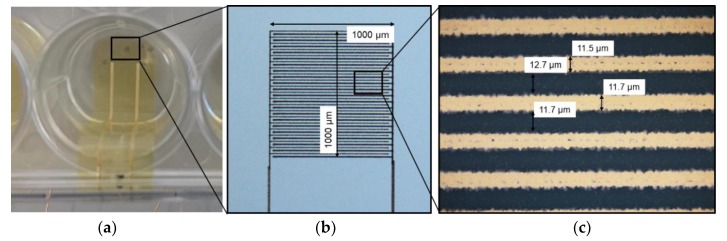
(**a**) Top view of the printed gold electrode (square) on flexible polyimide within the well of a standard 12-well cell culture plate; (**b**) microscopic view of the uncovered gold interdigital electrode with a printed thickness of approx. 300 nm; (**c**) line width and line space of the electrode fingers.

This type of flexible setup, comprising nanoparticle inks and polyimide substrates, are commonly used for printed electronics and can withstand typical bending experiments of 100,000 bending cycles with bending radii of less than 5 mm [30]. The flexible setup was placed within a standard 12-well cell culture plate (Figure 1a). To prevent the foil from floating during the cell experiments, it was attached with biocompatible silicone (Sylgard 184, Dow Corning, Midland, MI, USA) to the well bottom. Figure 1c shows the printing quality, especially the rough edges caused by the electrohydrodynamic inkjet process on a dielectric polyimide layer with a line pitch of approximately 24 µm.

### 2.2. Nanosecond Pulse Generator

**Figure 2:**
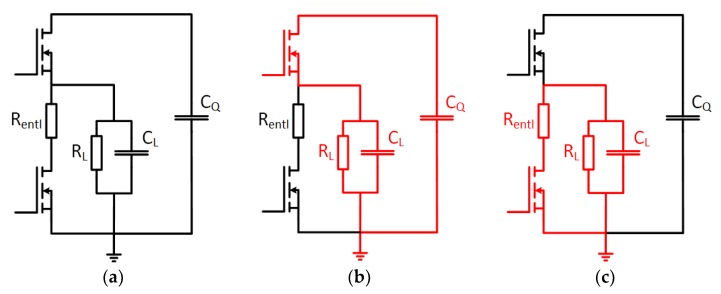
Circuit schematic of the operation modes of the bidirectional switch: (**a**) Bidirectional switch comprising two RF-MOSFETs, a discharge resistor (R_entl_), the load resistor (R_L_), load capacitance (C_L_) and the capacitor for power supply (C_Q_); (**b**) current flow during the nanosecond pulse; (**c**) current flow while discharging the load (R_L_).

A circuit for the electroporation experiments was designed and developed to generate rectangular pulses with 30–90 ns pulse width and 0–180 V amplitude. The circuit consists of a high-speed signal generator and a bidirectional switch, separated by an isolator circuit (MAX14130-FAEE+, Maxim Integrated, San Jose, CA, USA). The signal generator provides two control signals for the two RF-MOSFETs (DE150-102N02A, IXYS) of the bidirectional switch. One signal enables the rectangular pulse to deliver the high electric field at the electrode, while the second signal is required to discharge the electrode after pulsing. A dual gate driver (NCP81075, ON Semiconductor, Semiconductor Components Industries, LLC., Pheonix, AR, USA) provides switching energy for the two RF-MOSFETs. Figure 2 shows the operation modes of the bidirectional switch, which is responsible for the high voltage pulse delivery to the output. The demonstrated load resistor (R_L_) and capacitor (C_L_) represent where the electrode will be connected.

**Figure 3:**
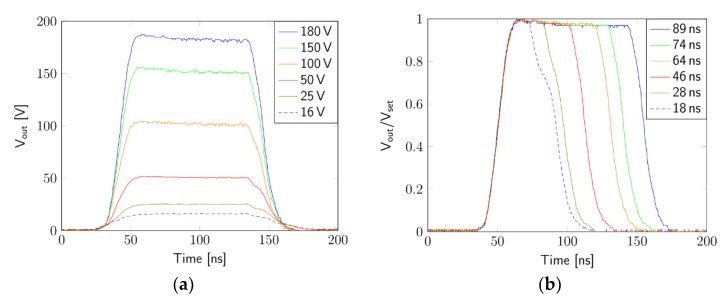
Measurement of the output signal: (**a**) different voltages with a set pulse length of 89 ns; (**b**) different pulse widths.

The output signal was measured at a sampling rate of 1 GSa/s with a Rhode & Schwarz TM 2054 4-channel oscilloscope with RTM-ZP10 test probes, providing bandwidths up to 500 MHz. To evaluate the load dependency of the circuit’s pulse generation, different measurements were made with various resistive and resistive-capacitive dummy loads connected to the output (1 kΩ, 1 kΩ parallel 10 pF, 500 Ω parallel 20 pF and 330 Ω parallel 30 pF), which showed no significant difference in the measured signal, even without separate impedance matching arrangements. The loads were chosen to meet the expected electrode impedance within the cell medium. Figure 3a shows the quality of the output signal at different voltages with the same pulse width.

Figure 3b shows the quality of the pulses at different pulse widths (measured at 90% of maximum value). The graph shows that the 18-ns pulse looks less accurate due to the gate driver’s limitation of 30 ns pulse width.

### 2.3. Experimental Setup for Electroporation

**Figure 4:**
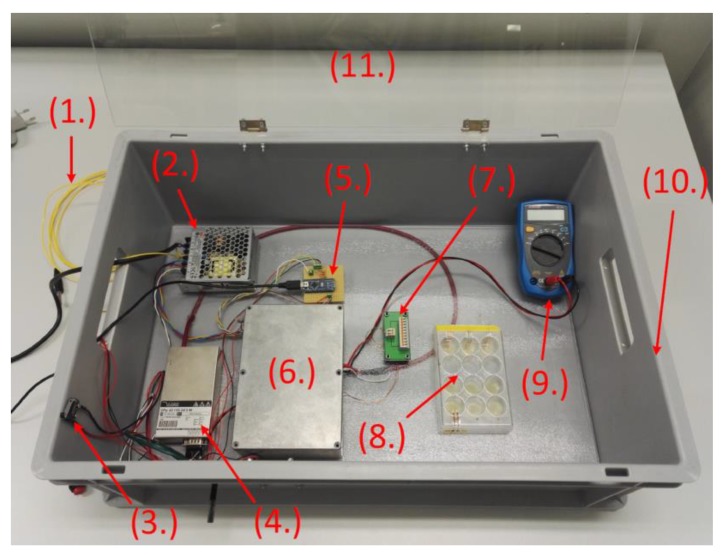
Experimental setup for electroporation experiments: (1) Grounding line, (2) Power supply, (3) Safety switch, (4) HV DC/DC Converter, (5) Arduino Nano, (6) Circuit board within the grounded metal case, (7) Rewiring board, (8) Cell culture plate, (9) Voltmeter, (10) Protection casing, (11) Protection lid.

The experimental setup was built in a protective polymer case, which comprises of components that securely perform the electroporation experiments (Figure 4). The high voltage DC/DC-converter (DPS Mini, iseg Spezialelektronik GmbH, Radeberg, Germany) converts 24 V input voltage into a variable output voltage between 0 V and 6 kV with a maximum output current of 1 mA. Arduino NANO provides the input signal for the signal generator and controls various components on the circuit board. A standard voltmeter (Voltcraft VC120) was used to monitor and set the voltage of the DC/DC converter prior to the pulses. The designed signal generator and the controlling circuit for the output pulses were located on a printed circuit board, which was mounted in a metal case. The cell culture plate was placed in the box shortly before the experiments. The wires from the flexible electrodes were connected to the rewiring board.

### 2.4. Cell Culture and Exposure to Electric Fields

**Figure 5:**
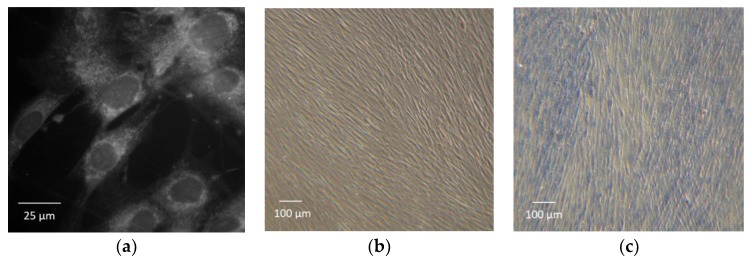
Image of vital fibroblasts (HFFF2) in: (**a**) detailed view and (**b**) lower magnification for demonstration of the typical elongated shape; (**c**) Image of dead fibroblasts dyed with trypan blue at lower magnification (10×).

Human fibroblast cells (cell line HFFF2) were chosen for the cell experiments due to their known robustness and good adherence. Figure 5a shows a high-resolution picture of the fibroblasts taken by a Zeiss Axio Imager.Z1 with a 40x dipping objective (BIOTEC, TU Dresden, Germany). The cells were dyed with cyanine (Cy3) and evaluated with a filter F46-004 (AHF Analysentechnik AG, Tübingen, Germany) and a Zeiss AxioCam MRm camera. Figure 5b shows the typical elongated shape of the fibroblasts with 10x objective, taken with available equipment for the nsPEF experiments (CK40 transmitted-light microscope, Olympus Corporation, Shinjuku, Tokyo, Japan; PowerShot G9 digital camera, Canon Inc., Ota City, Tokyo, Japan). The flexible polyimide substrate is well known for its biocompatibility and has been proven as such in cell contact studies in previous publications [31]. The cell culture plates, including the electrodes, were sterilized using UV light for 30 min before adding 200,000 cells and 2 mL cell culture medium (Dulbecco’s Modified Eagle’s Minimum Essential Medium (DMEM) mixed with Ham’s F12 in ratio 1:2 (DMEM/F12), 10% fetal calf serum (FCS)) to every well. The cells were in the cell medium throughout the experiment. The electrical conductivity of the medium was 11 mS/cm and determined using the conductivity electrode SevenMulti S70 (Mettler Toledo, Columbus, OH, USA). The cells in direct contact with the electrodes showed 100% confluence after four days of incubation in a humidified atmosphere at 37 °C and 5% CO_2_ content.

The test was performed two times. During each test, two active electrodes (applying pulses) and one passive electrodes (no pulses) were utilized. The electrode without pulses was used for reference, and cells growing on the blank well bottom helped double-check overall cell behavior during the tests.

The nsPEF treatment was performed with only one set of electrical parameters: 50 pulses with a pulse width of 45 ns and pulse amplitude of 180 V, corresponding to an electric field strength of approximately 100 kV/cm. The electric field strength was estimated by the parallel-plate capacitor equation, neglecting the inhomogeneous parts of interdigital electrodes. No parameter variation was done, as this first test only verified the feasibility of the printed micro-electrodes. The cells were further incubated and examined after pulse treatment, using light-microscopy at different time intervals.

Onsite resistance measurement (in addition to optical inspection) with a multimeter via the electrodes and the cell medium turned out as an indicator of cell confluency. An electrode with low percentage of cell confluence showed resistance in the lower kiloohm range. However, a confluent, cell multilayer showed resistance in the megaohm range, indicating that the cell membrane was not conductive. This highlights the fact that ohmic resistance of the electrodes themselves can be neglected if pulses are applied in this case.

A color test using trypan blue (TB) was performed on some electrodes to determine the permeabilization effects of nsPEF on the cell membrane [32]. Trypan blue is typically used to color dead cells among vital ones [33]. The dye naturally does not pass the membranes of most cells, due to its relatively large molecule size—about 0.6 nm. However, in the case of increased membrane permeability or damage, TP is able to cross the membrane and enter the cells. Figure 5c shows fibroblasts as a reference, treated with methanol and dyed with TP. For this, a solution of cell medium and 0.5% trypan blue was added to the cells and removed 5 min after. Finally, careful rinsing of the wells several times with a cell medium results in only the trypan blue being left behind in the cells.

## 3. Results

**Figure 6:**
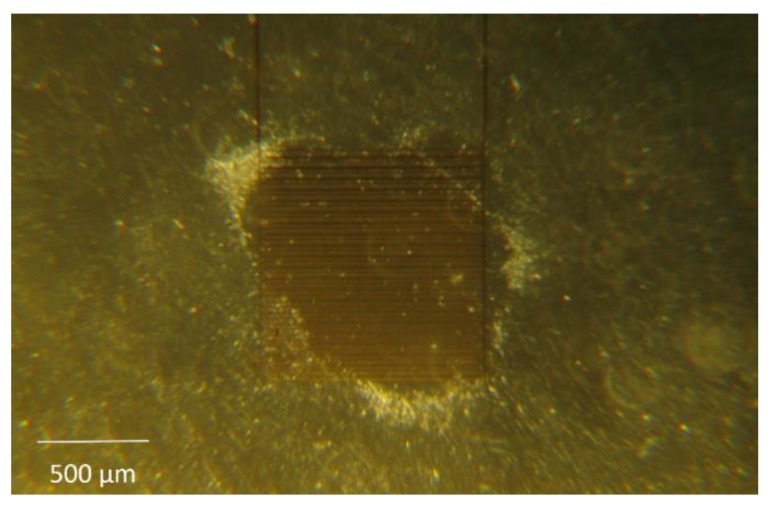
Exemplary picture of a flexible, printed electrode (center) after nsPEF treatment. The cell layer around the electrode is still as dense as before pulsing. However, most of the interdigital electrode area is almost free of fibroblasts and cell clusters formed at the electrode’s edge.

The fibroblasts on pulsed electrodes showed eventual similar behavior due to same pulse parameters after nsPEF treatment. Figure 6 shows an electrode free of cells after nsPEF treatment; the area surrounding the electrode stayed the same as before the treatment.

Although pulses of the same parameters were applied and the outcome of the nsPEF treatment led to similar results, the course of the experiments with different electrodes varied. Hereafter, the results of two electrodes are described in detail to demonstrate the observations.

**Figure 7:**
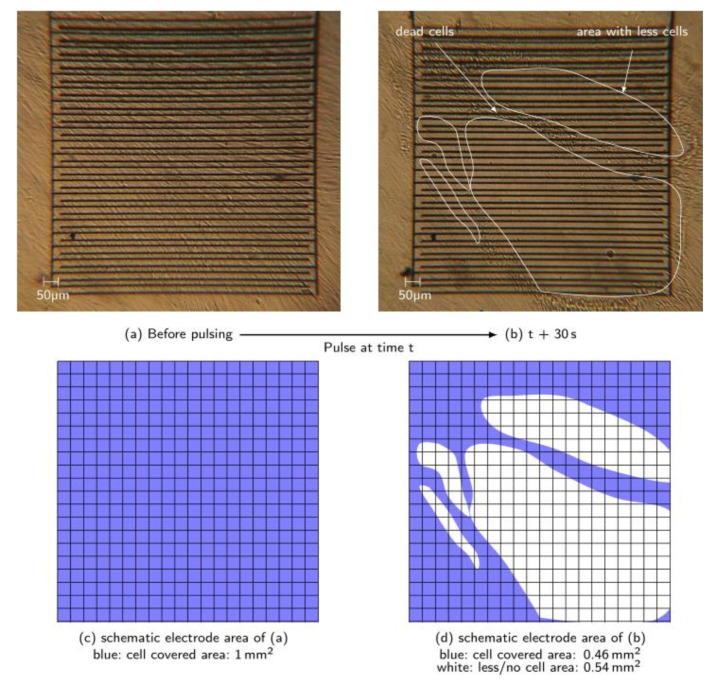
(**a**) Picture of the electrode with 100% cell confluence before nsPEF treatment; (**b**) picture of the electrode 30 s after applying the pulses. Cells were still partially adhered and partially already detached, which results in areas with significantly less cells. Vital cells were still observed at the edges of the electrode; (**c**) and (**d**) schematic electrode area (1 × 1 mm^2^, grid dimensions 50 × 50 µm^2^) with high density cell coverage (blue) and zones where less or no cells cover the electrode (white) after pulsing.

Figure 7a shows the dense cell multilayer with 100% confluence before the pulses are applied (Figure 7c). About 30 s after the nsPEF treatment, large areas of the electrode were almost free of cells (Figure 7b,d). Those areas with noticeably less cells correspond approximately to 54% of the overall electrode area and was pixel-analyzed using software ImageJ by Wayne Rasband (National Institutes of Health (NIH), Bethesda, MD, USA). The blue color in Figure 7c,d represents areas where cells are on top of the electrode. The white zones represent parts of the electrode with no or few cells on the electrode. The exact time period between the pulses and the image was not determined, because 30 s corresponds to the time needed to position the wellplate on the microscope. Within the marked area, individual cells were still present and attached, but clearly, the vast majority was detached and vanished. Furthermore, a band of cumulative detached cells moves over the electrode (Figure 7b).

**Figure 8:**
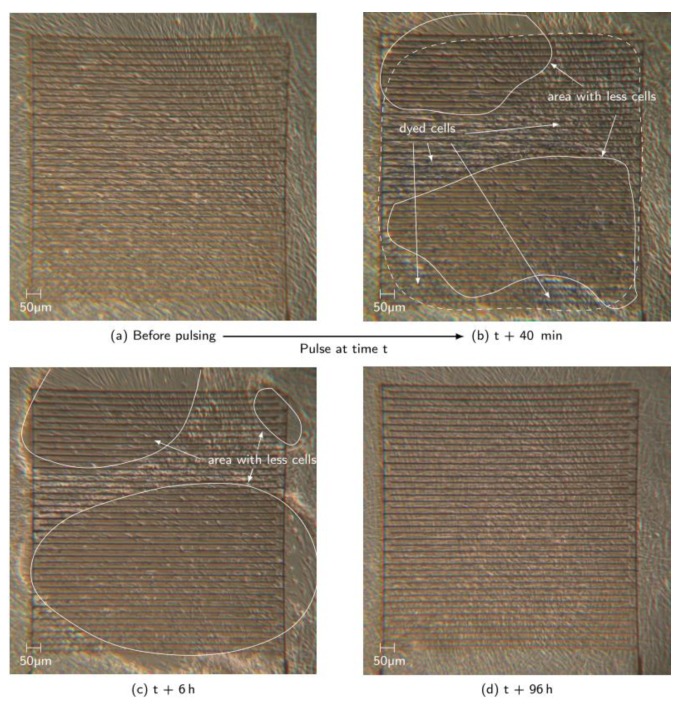
(**a**) Picture of the electrode before nsPEF treatment with vital cells; (**b**) picture of the electrode after trypan blue test; (**c**) increased area with less cells; (**d**) fully recovered cell layer on the electrode 96 h after nsPEF treatment.

However, the area surrounding the electrode was still covered with vital cells. This was tested with a TB test (not documented), and also optically evaluated by monitoring cell growth for several hours after. The detached cells’ band was dyed blue. The electrode was re-covered due to cell growth after sufficient time—demonstrated more clearly in Figure 8.

**Figure 9:**
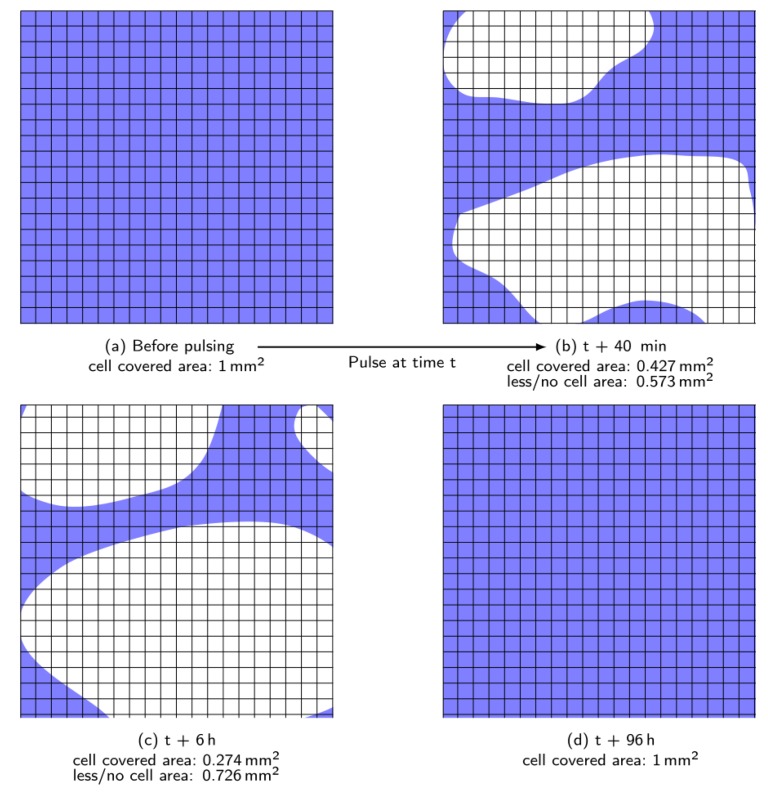
Schematic picture showing only the electrode area (1 × 1 mm^2^, grid dimensions 50 × 50 µm^2^) corresponding to Figure 8. (**a**) and (**d**) showing fully cell covered electrode areas (blue) before and 96h after pulsing; (**b**) shows decreasing cell covered area and increasing area without or less cells in white; (**c**) demonstrates increased white area after about 5 h compared to (**b**) and therefore even fewer cells remain on the electrode.

Figure 8 shows an experiment with the same pulsing parameters on a different electrode. The time period between the pulses and the first occurring larger area with clearly noticeable detached cells was much longer (ca. 40 min). First (directly after the nsPEF treatment), individual cells disappeared, contracted, and then swelled. Yet, those reactions were barely noticeable. For that reason, a TB test was performed to distinguish vital cells from dead cells. Large areas on the electrode were dyed blue, as shown within the dashed line in Figure 8b. In this particular case, it does not necessarily mean that all the cells were dead, only that the TB molecules were able to diffuse into the cells. Thus, electropermeabilization may have happened. Additionally, the area without cells first becomes larger with increasing time after application of the pulses, before it gets smaller again, due to regrowth of the surviving cells. Figure 9 shows, corresponding to Figure 8, only an area of the electrode size of 1 × 1 mm^2^. Figure 9b,c show an increase of approximately 15% of the area, where no or less cells were seen on different zones on the electrode. Those pictures showing the first period of increasing area until it reaches its maximum extent (approximately 73%) 6 h after the pulsing. Figure 8b,c shows that the TB dye color is not noticeable. This might be a sign that the membrane is still permeable or the cells have died, thus diffusing TB molecules out of the cell. Yet, cell clusters moved from the left to the right over the electrode, indicating dead cells. Dead fibroblasts usually detach from the surface, which was clearly noticeable when the well plate was gently moved. However, the exact point of death needs further investigation. From 6 h after application onwards, the electrode started to be re-covered with cells, up to 96 h after the pulsing (Figure 8d).

## 4. Conclusions

A novel miniaturized flexible microelectrode was additively manufactured with gold nanoparticle ink on polyimide substrate with a line and spacing width down to approximate 10 µm. This enables a possible future integration of the electrode within microsystems for clinical trials when connected to an approved parameter-matching electroporator. However, electrical and mechanical reliability of the dependency of the printed fine lines has to be further investigated. The developed electrical circuit showed very little load dependency and was able to transmit pulses to the flexible miniaturized electrode with the intended pulse waveforms.

Furthermore, the performed nsPEF experiments using the printed, biocompatible and flexible microelectrode demonstrated the application of sufficient electric field strength to induce distinct reactions on fibroblasts. After the pulsing, the cell count on almost the whole electrode area was significantly reduced. The results show strong evidence of electropermeabilization of the cell membranes and clear cell movement off the electrode after pulsing. The performance of TB test, emphasized membrane permeabilization indicators, such as swelling of the cells. The known toxicity of TB during longer incubations [32] might have contributed to the cell death as well, but has not been investigated. However, TB was able to pass the membrane, which indicated relatively large pores formed (at least of the size of TB). Hence, the electropermeabilization effect might have caused fast death of the cells as well [32]. The background for cell death needs further characterization, together with the proposed setup. Future spectroscopic investigations of the dye intensity might give more clarity, as well as the use of a different test essay with various pulse parameters.

However, the fact that not all electrode areas are free of cells and the variation in results needs further investigation, especially the variance in time period between pulse application and noticeable cell reaction between seconds and minutes after pulse application. This can be better understood by investigating the dependency of the electric field strength and form on cell coverage after the nsPEF treatment using these electrodes. Additionally, the dose of the electric field directly at the cells needs to be defined. A simulation of the interdigital electrode setup, based on a cell-resistance model, needs to be performed to describe the varying cell parameters, or as an alternative, further experiments should be conducted with different cell confluences. The electrode showed good first results on electropermeabilization when applying nanosecond pulses. For future medical applications such as ECT, the electrode has to be tested with the more typically used 100 µs pulses to prove further eligibility.

The proposed setup should be implemented in a real-time microscopic camera environment to add to this work and study cell behavior with different pulsing parameters.
